# Efficacy of Sertraline Combined With Intestinal Microecological Therapy in Adolescents With Moderate Depression and Suicidal Ideation and Its Effects on Serum Inflammatory Factors

**DOI:** 10.62641/aep.v54i1.2127

**Published:** 2026-02-15

**Authors:** Lei Zhang, Dongrong Zhao, Jiayu Huang, Sha Liu, Naihong Xu

**Affiliations:** ^1^First School of Clinical Medical, Gansu University of Chinese Medicine, 730000 Lanzhou, Gansu, China; ^2^Department of Mental Health, Gansu Provincial Hospital, 730000 Lanzhou, Gansu, China; ^3^First Clinical Medical College, Northwest Minzu University, 730030 Lanzhou, Gansu, China

**Keywords:** adolescent depression, suicidal ideation, sertraline, intestinal microecology, inflammatory factor, gut-brain axis

## Abstract

**Background:**

Adolescents with moderate depression and suicidal ideation constitute a high-risk psychiatric population. Major depressive disorder with suicidal ideation in this age group is a disabling psychiatric disorder. Current selective serotonin reuptake inhibitor treatments are limited by their low efficacy rates (approximately 50%–60%) and delayed onset of action. Informed by the gut–brain axis theory, this study aimed to evaluate the synergistic efficacy and anti-inflammatory mechanisms of sertraline combined with a *Bacillus subtilis* probiotic preparation in this high-risk population.

**Methods:**

This retrospective cohort study included 160 adolescents meeting International Classification of Diseases 10th Revision diagnostic criteria were identified and categorised into either monotherapy (sertraline) or combination therapy (sertraline + probiotics) groups. Over a 12-week treatment period, clinical symptoms were assessed using the Hamilton Depression Rating Scale, Hamilton Anxiety Rating Scale and Beck Scale for Suicide Ideation-Chinese Version, and serum inflammatory factors, namely, interleukin-6 (IL-6), interleukin-1β (IL-1β), tumour necrosis factor-α (TNF-α) and C-reactive protein (CRP) and peripheral blood inflammatory ratios, namely, platelet-to-lymphocyte, neutrophil-to-lymphocyte and monocyte-to-lymphocyte ratios, were measured.

**Results:**

The proportion of patients with severe depression was significantly reduced in the combination group (1.25% vs*.* 7.5%, *p* = 0.004), and anxiety symptoms showed significant improvement (severe anxiety proportion: 1.25% vs. 11.25%, *p* = 0.008). Biomarker analysis revealed significantly reduced levels of IL-6 (*p* = 0.007), IL-1β (*p* = 0.002), TNF-α (*p* = 0.005) and CRP (*p* = 0.001) in the combination group, and IL-6 and CRP showed strong positive correlations with depression scores (*r *= 0.35–0.39).

**Conclusions:**

This study confirms that modulating the intestinal smicroecology can enhance antidepressant efficacy by reducing neuroinflammation.

## Introduction

Major depressive disorder (MDD) is a psychiatric disorder characterised primarily by persistent depressed mood, loss of interest and cognitive dysfunction, and it has emerged as a global public health concern [1]. According to statistics from the World Health Organization, approximately 350 million people worldwide are affected by depression, which is projected to become the leading cause of global disease burden by 2030 [2]. Of particular concern is the increasing annual incidence of adolescent depression. Approximately 24.6% of Chinese teenagers suffer from depression, and 7.4% suffer from severe depression [3].

Adolescents are in a critical period of physical and mental development. Depression severely affects their academic performance and social functioning, and adolescent patients are more prone to suicidal ideation and behaviour than adult patients [4]. Approximately 38.2% of adolescent patients with depression experience suicidal ideation [5]. Therefore, exploring effective treatment strategies for adolescent depression accompanied by suicidal ideation holds considerable clinical and societal importance. To date, selective serotonin reuptake inhibitors (SSRIs) remain the first-line pharmacological treatment for adolescent depression, and sertraline has been widely used because of its confirmed efficacy and high safety profile [6]. Notably, evidence suggests that combination therapies (e.g., integrating pharmacotherapy with psychosocial interventions) often yield outcomes superior to those of monotherapy in this population [7]. Additionally, adolescents’ low tolerance to the side effects of medications restricts dose escalation and potential for improved efficacy [8]. In recent years, the development of the ‘gut–brain axis’ theory has provided new perspectives on the pathophysiological mechanisms of depression [9]. Building upon this understanding, clinical research has begun to explore intestinal microecological modulation as a complement to antidepressant pharmacotherapy. Intestinal microecological imbalance is closely related to the occurrence and development of depression [10]. The gut microbiota interacts with the central nervous system through neural, endocrine and immune pathways, affecting neurotransmitter synthesis, hypothalamic-pituitary-adrenal (HPA) axis function and neuroinflammatory responses [11]. This mechanistic understanding underpins the rationale for combining probiotics with antidepressants, and adjunctive therapy may enhance treatment response and improve certain depressive symptoms. However, existing evidence primarily stems from research conducted on general adult depression populations. Beneficial bacteria, such as *Lactobacillus* and *Bifidobacterium*, are considerably reduced in the intestines of patients with depression, whereas pathogenic bacteria relatively increase. This dysbiosis may contribute to the pathogenesis of depression by increasing intestinal permeability and promoting the release of inflammatory factors [12]. The inflammation hypothesis is one of the important theories explaining the pathological mechanisms of depression [13]. Patients with depression exhibit chronic low-grade inflammation, manifested by elevated levels of pro-inflammatory cytokines such as interleukin-6 (IL-6), interleukin-1β (IL-1β) and tumour necrosis factor-α (TNF-α) [14, 15]. These inflammatory factors can cross the blood-brain barrier, enter the central nervous system, activate microglia and induce indoleamine 2,3-dioxygenase activity, disrupting the normal activity of tryptophan-kynurenine metabolic pathways and ultimately affecting serotonin synthesis and triggering depressive symptoms [16]. Notably, patients with depression and suicidal ideation often have high inflammatory factor levels. This observation suggests that inflammatory responses play an important role in the neurobiological mechanisms of suicidal behaviour [17].

Despite these promising theoretical foundations, critical gaps remain in the current research landscape. Specifically, studies focusing on the adolescent population, which is a group with distinct neurodevelopmental and psychosocial characteristics, are lacking, and few studies have focused on adolescents with co-occurring depressive disorder and suicidal ideation. Furthermore, although the anti-inflammatory potential of probiotics is recognised, direct evidence linking their adjunctive use with changes in systemic inflammatory biomarkers in adolescent depression is scarce.

Intestinal microecology modulation informed by the aforementioned theoretical foundation is a novel therapeutic strategy for improving depressive symptoms. Probiotic preparations can restore gut microbiota balance, repair intestinal barrier function and reduce bacterial endotoxin translocation, thereby lowering systemic inflammation levels [18]. Additionally, certain probiotic strains can directly or indirectly participate in neurotransmitter synthesis. For example, *Lactobacillus* can produce γ-aminobutyric acid, and *Bifidobacterium* can affect tryptophan metabolism [19]. Probiotic adjuvant therapy can alleviate mood symptoms and cognitive function in patients with depression [20]. However, research on intestinal microecological preparations combined with antidepressants to treat adolescent depression, particularly for patients with suicidal ideation, remains limited.

This study aims to investigate the clinical efficacy of sertraline combined with *Bacillus subtilis* dual live bacteria enteric capsules in treating adolescents with moderate depression and suicidal ideation, to explore possible mechanisms from the perspective of inflammatory factors and to provide safe and more effective comprehensive treatment options for clinical practice. The present study addresses the aforementioned gaps by focusing on a specialised adolescent clinical sample, employing a specific probiotic formulation combined with first-line SSRI therapy and comprehensively evaluating outcomes not only in terms of core depressive and suicidal symptoms but also through a panel of serum inflammatory biomarkers. This approach allows for an integrated assessment of clinical efficacy and potential neuroimmunological mechanisms.

## Materials and Methods

### Study Participants

A single-centre retrospective cohort study was conducted, and the protocol was approved by the ethics committee of Gansu Provincial Hospital (No. 2025-439). The study was conducted in accordance with the principles of the Declaration of Helsinki. Adolescents with moderate depression and suicidal ideation who were admitted to the mental health department’s outpatient and inpatient units at Gansu Provincial Hospital from January 2024 to April 2025 were recruited as study subjects.

#### Inclusion Criteria

(1) Age of 14–18 years (any gender).

(2) Meeting International Classification of Diseases 10th Revision (ICD-10) diagnostic criteria for MDD and having a baseline Hamilton Depression Rating Scale-24 (HAMD-24) score of ≥20 (indicating at least moderate severity).

(3) Presence of suicidal ideation on initial Beck Scale for Suicide Ideation (BSI-CV) with a baseline score of ≥6, indicating clinically significant suicidal ideation.

(4) Voluntarily participation (by patients or legal guardians) and informed consent.

#### Exclusion Criteria

(1) Presence of other psychiatric disorders (such as schizophrenia, bipolar disorder and generalised anxiety disorder).

(2) Use of probiotic products ≥7 days within the past month.

(3) Use of antibiotics, corticosteroids, non-steroidal anti-inflammatory drugs or immunomodulators within the past 3 months.

(4) Allergy or intolerance to medications.

### Study Methods

#### Basis for Study Grouping

On the basis of treatments received as documented in prescription records, eligible patients were divided into two groups: combination group (*n *= 80), which include patients who had been prescribed sertraline hydrochloride concurrently with *B. subtilis* dual live bacteria enteric capsules, and monotherapy group (*n* = 80), which included patients who had been prescribed sertraline hydrochloride alone without concomitant probiotic use during the study period.

#### Sample Size Estimation

Sample size was estimated using PASS 22.0 software (IBM Corporation, Armonk, NY, USA). The following parameters were set: statistical power (1-β) = 0.9, two-sided test, significance level (α) = 0.05. The primary outcome measure was the HAMD-24 score. The results of the pilot study show that the post-treatment HAMD-24 score mean (*µ*_t_) for the ‘Antidepressant + Gut Microbiota’ group was 8.83 and the mean (*µ*_c_) for the ‘Antidepressant’ group was 11.09. The common standard deviation (σ) was 4.28. With *t*_α/2_ = 1.97569, *t*_β_ = 1.28715 and allocation ratio (*k*) = 1, the sample size calculation formula yielded a required sample size of 77 subjects for the control group (Antidepressant) and *nt* = *k* ×*n*_c_ = 77 subjects for the combination therapy group (Antidepressant + Gut Microbiota), resulting in a total sample size of 154. The actual calculated power for this sample size was 0.90245. A total of 160 patients (80 per group) were finally included. This number exceeded the minimum estimated sample size, ensuring that the study had adequate statistical power.

$$n_{c}=\frac{{\left(t_{\alpha /2}+t_{\beta }\right)}^{2}\times \sigma^{2}\times \left(1+1/k\right)}{{\left(\mu_{t}-\mu_{c}\right)}^{2}}$$

Here, *n*_c_ is the sample size of the Antidepressant group, *k* is the sample size ratio of the Antidepressant + Gut Microbiota group to the Antidepressant group, *µ*_t_ is the mean of the Antidepressant + Gut Microbiota group, *µ*_c_ is the mean of the Antidepressant group, σ is the standard deviation of the Antidepressant + Gut Microbiota group, *t*_α/2_ is the upper α/2 quantile of the t-distribution and *t*_β_ is the upper β quantile of the t-distribution.

#### Intervention Protocol

Treatment Group (*n* = 80): Sertraline hydrochloride (Zhejiang Jingxin Pharmaceutical Co., Ltd., Shaoxing, Zhejiang, China, 50 mg/tablet, National Medicine Approval Number: H20051076) combined with *B. subtilis* dual live bacteria enteric capsules (Beijing Hanmi Pharmaceutical Co., Ltd., Beijing, China; National Medicine Approval Number: S20030087, 250 mg/capsule). The starting dose of sertraline was 50 mg per day, increased to 100 mg per day on day 4 and further increased to 150 mg/per day on day 7 depending on the patients’ conditions. The drug was administered twice daily (morning and evening). *B. subtilis* dual live bacteria enteric capsules (500 mg) were administered three times daily and taken 30 minutes after meals.

Control Group (*n* = 80): Patients were prescribed sertraline hydrochloride alone without the concomitant use of any probiotic preparations during the study period. The dosage followed the same regimen as the treatment group.

Both groups received treatment for 12 weeks: 2 weeks of inpatient observation and 10 weeks of outpatient follow-up. During treatment, the use of other antidepressants, antipsychotics, mood stabilisers and probiotic products was prohibited.

#### Outcome Measures

Primary Efficacy Indicators: (1) Depression severity is assessed using the HAMD-24, which includes 24 items evaluating depressive symptoms, such as mood, guilt, insomnia and somatic complaints. Total scores range from 0 to 76 and are interpreted as follows: <8 = no depression, 8–19 = mild, 20–34 = moderate and ≥35 = severe. The Chinese version demonstrates high internal consistency (Cronbach’s α = 0.92) and has been validated in adolescent populations [21]. (2) Suicidal ideation is assessed using the BSI-CV. This 19-item scale measures the presence and intensity of suicidal thoughts and intent. Items are rated from 0 to 2, yielding total scores from 0 to 38. High scores indicate high suicide risk (a score of ≥6 is commonly used as a clinical threshold). The Chinese version has shown strong reliability (Cronbach’s α = 0.86) and good convergent validity in adolescent clinical samples [22].

Secondary Efficacy Indicators: (1) Anxiety symptoms are assessed using the Hamilton Anxiety Rating Scale (HAMA), which is a 14item clinicianrated scale measuring psychic and somatic anxiety symptoms. Each item is scored from 0 (not present) to 4 (severe), yielding a total score ranging from 0 to 56. Severity is classified as follows: <7 = no anxiety, 7–17 = mild, 18–24 = moderate and >24 = severe. The Chinese version demonstrates high internal consistency (Cronbach’s α = 0.915) and has been validated in adolescent clinical samples. (2) Serum inflammatory factors: IL-6, IL-1β, TNF-α and C-reactive protein (CRP). (3) Peripheral blood inflammatory indicators: neutrophil-to-lymphocyte ratio (NLR), platelet-to-lymphocyte ratio (PLR), monocyte-to-lymphocyte ratio (MLR).

In accordance with the standard clinical protocol documented in the medical records, blood samples for inflammatory factor measurement were routinely collected in the morning (typically between 7:00 and 9:00 AM) after an overnight fast. The serum levels of IL-6, IL-1β and TNF-α were measured in the hospital laboratory and with commercially available enzyme-linked immunosorbent assay kits (R&D Systems, Minneapolis, MN, USA). High-sensitivity CRP was determined with a latex-enhanced immunoturbidimetric assay on an automated clinical chemistry analyser (Cobas c 501, Roche Diagnostics). Peripheral blood cell counts for calculating NLR, PLR and MLR were determined from routine complete blood count (CBC) reports generated using a Sysmex XN-1000 haematology analyser. All laboratory procedures followed the standard operating protocols of the hospital’s clinical laboratory.

### Statistical Analysis

All statistical analyses were performed using SPSS software (version 26.0, IBM Corp., Armonk, NY, USA). The normality of continuous variables was first assessed using the Shapiro-Wilk test. When the data followed a normal distribution (*p* ≥ 0.05), they were presented as mean ± standard deviation (SD). Betweengroup comparisons were conducted using independentsamples *t*-tests. When the normality assumption was violated (*p* < 0.05), data were expressed as median with interquartile range (25th–75th percentile), and betweengroup comparisons were performed using the Mann-Whitney *U* test. Categorical variables were summarised as number (percentage) and compared using the chi-square (χ^2^) tests. Changes in continuous scale scores (HAMD-24, HAMA and BSI-CV) were analysed using analysis of covariance (ANCOVA), with baseline scores included as a covariate. The Mann–Whitney *U* test was employed for ordinal categorical variables (e.g., depression and anxiety severity categories). The relationships between psychological scale scores and inflammatory factors were explored through Spearman correlation analysis. A two-tailed significance level of 0.05 was adopted for all statistical tests.

## Results

### Baseline Characteristics Comparison

A total of 160 patient records were included (80 in each group). No statistically significant differences in age, gender, disease duration, baseline HAMD-24 scores and BSI-CV scores (*p* > 0.05) were found between groups, indicating comparability. During the data collection period, Six patients (7.5%) in the treatment group and eight patients (10.0%) in the control group had incomplete data for analysis (Table 1). The primary reason for data incompleteness was loss to follow-up documentation. No significant difference was found between groups (*p* = 0.574).

**Table 1.** **Baseline characteristics**.

		Treatment group	Control group	*t*/χ^2^	*p*
Gender				0.130	0.719
	Male	20 (25.00)	22 (27.50)		
	Female	60 (75.00)	58 (72.50)		
Age (years)				1.223	0.874
	14	14 (17.50)	16 (20.00)		
	15	15 (18.75)	17 (21.25)		
	16	23 (28.75)	17 (21.25)		
	17	15 (18.75)	16 (20.00)		
	18	13 (16.25)	14 (17.50)		
Disease duration (months), mean ± SD		8.51 ± 3.20	8.83 ± 3.52	0.566	0.572
Baseline HAMD-24, mean ± SD		32.14 ± 5.21	31.72 ± 4.93	0.501	0.617
Baseline HAMA, mean ± SD		25.44 ± 4.50	24.91 ± 4.84	0.680	0.498
Baseline BSI-CV, mean ± SD		18.34 ± 3.73	17.92 ± 4.01	0.657	0.512
Dropout				0.316	0.574
	Yes	6 (7.50)	8 (10.00)		
	No	74 (92.50)	72 (90.00)		

HAMD-24, Hamilton Depression Rating Scale-24; HAMA, Hamilton Anxiety Rating Scale; BSI-CV, Beck Scale for Suicide Ideation; SD, standard deviation.

### Clinical Efficacy Comparison

#### Comparison of Scale Scores

The continuous scale scores (HAMD-24, HAMA and BSI-CV) of the groups after treatment were compared using ANCOVA, and post-treatment scores were the dependent variables, the treatment group was the independent variable and baseline scores were the covariates. After adjusting for baseline scores, ANCOVA revealed that the combination therapy group showed significantly greater reduction in HAMD-24 (*F* = 10.78, *p* = 0.001), HAMA (*F* = 7.84, *p* = 0.006) and BSI-CV scores (*F* = 15.14, *p* < 0.001) than the monotherapy group, demonstrating the statistically superior efficacy of the combined treatment in alleviating depressive, anxiety and suicidal ideation symptoms (Table 2).

**Table 2.** **Comparison of depression, anxiety and suicidal ideation scale scores before and after intervention in both groups (Mean ± SD)**.

Scale	Groups	Baseline	Post-Treatment	Adjusted Mean Difference (95% CI)	*p*-value
HAMD-24	Treatment group	32.1 ± 5.2	7.8 ± 4.1	−2.8 (−4.2, −1.4)	0.001
	Control group	31.7 ± 4.9	10.5 ± 5.0	Ref	
HAMA	Treatment group	25.4 ± 4.5	6.9 ± 3.8	−2.2 (−3.8, −0.6)	0.006
	Control group	24.9 ± 4.8	9.1 ± 4.3	Ref	
BSI-CV	Treatment group	18.3 ± 3.7	4.2 ± 2.9	−2.6 (−3.8, −1.4)	<0.001
	Control group	17.9 ± 4.0	6.8 ± 3.5	Ref	

HAMD-24, Hamilton Depression Rating Scale-24; HAMA, Hamilton Anxiety Rating Scale; BSI-CV, Beck Scale for Suicide Ideation; CI, confidence interval.

#### Improvement in Depression Severity

Distribution differences in depression severity between the two groups were assessed using the Mann–Whitney U test, and the ordinal categorical variable was analysed (none, mild, moderate and severe). The distribution of depression severity in the combination therapy group was significantly better than that in the monotherapy group (*Z* = –2.894, *p* = 0.004). Specifically, the proportion of patients without depression was higher in the combination group (73.75%) than in the monotherapy group (62.5%), and the proportions of patients with moderate and severe depression (combination group: 3.75% & 1.25%; monotherapy group: 13.75% & 7.5%) were significantly lower (Table 3).

**Table 3.** **Depression severity distribution**.

	Treatment group	Control group	Z	*p*
No depression	59 (73.75)	50 (62.50)	−2.894	0.004
Mild	17 (21.25)	13 (16.25)		
Moderate	3 (3.75)	11 (13.75)		
Severe	1 (1.25)	6 (7.50)		

#### Improvement in Anxiety Symptoms

Based on the ordinal categorical outcomes of the HAMA scale, the Mann-Whitney U test confirmed that the distribution of anxiety severity in the combination therapy group was significantly better than that in the monotherapy group (*Z* = –2.651, *p* = 0.008). The proportion of patients without anxiety symptoms was higher in the combination group (35.00%) than in the monotherapy group (22.50%), and the proportion of patients with severe anxiety was much lower in the combination group (1.25%) than in the monotherapy group (11.25%) (Table 4). These results are consistent with the findings from the analysis of continuous HAMA scores.

**Table 4.** **Anxiety severity distribution**.

	Treatment group	Control group	*Z*	*p*
No anxiety	28 (35.00)	18 (22.50)	−2.651	0.008
Mild	39 (48.75)	43 (53.75)		
Moderate	12 (15.00)	10 (12.50)		
Severe	1 (1.25)	9 (11.25)		

### Changes in Serum Inflammatory Factors

**Fig. 1.**
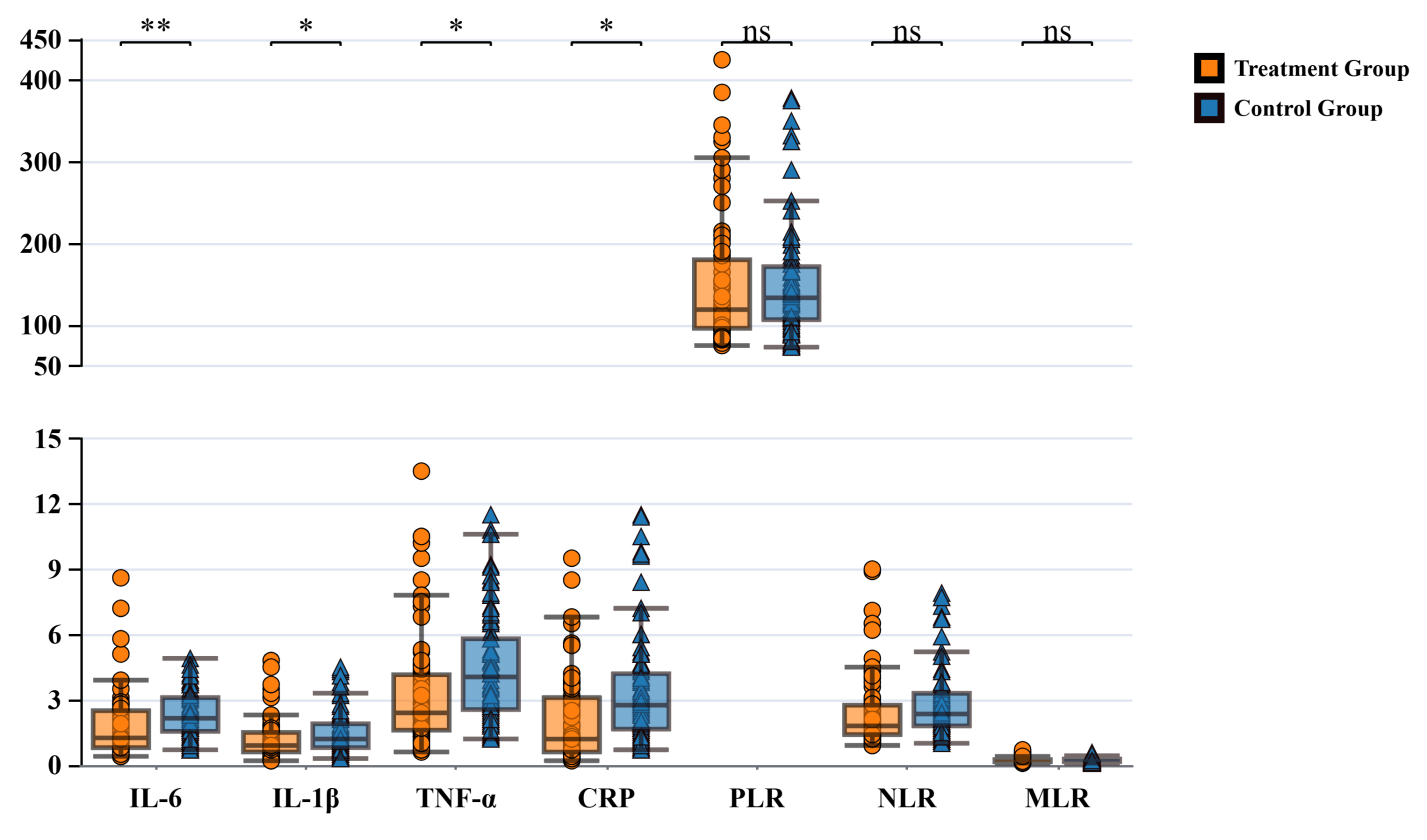
**Comparison of serum inflammatory factors and peripheral blood inflammatory ratios between groups after treatment**. IL-6, interleukin-6; IL-1β, interleukin-1β; TNF-α, tumor necrosis factor-α; CRP, C-reactive protein; PLR, platelet-to-lymphocyte ratio; NLR, neutrophil-to-lymphocyte ratio; MLR, monocyte-to-lymphocyte ratio; ns, not significant. *p < 0.05, **p < 0.01.

At baseline, no statistically significant differences in serum levels of IL-6, IL-1β, TNF-α and CRP were found between the groups (all *p* > 0.05), indicating comparable inflammatory status prior to treatment. After 12 weeks of treatment, serum inflammatory factor levels decreased to varying degrees in both groups, but the decrease was more significant in the treatment group. Intra-group comparisons revealed that the levels of IL-6 (*p* = 0.007), IL-1β (*p* = 0.002), TNF-α (*p* = 0.005) and CRP (*p* = 0.001) decreased significantly from baseline in the combination group. In the monotherapy group, only IL-6 (*p* = 0.014) and CRP levels (*p* = 0.025) showed significant reductions, while the decreases in IL-1β (*p* = 0.102) and TNF-α levels (*p* = 0.113) were not statistically significant. IL-6 levels in the treatment group were 1.8 ± 1.5 pg/mL, significantly lower than 2.3 ± 1.0 pg/mL in the control group (*p* = 0.008); IL-1β levels were 1.2 ± 0.9 pg/mL, lower than 1.5 ± 1.0 pg/mL in the control group (*p* = 0.034); TNF-α levels were 3.4 ± 3.0 pg/mL, lower than 4.4 ± 2.5 pg/mL in the control group (*p* = 0.022); CRP levels were 2.4 ± 3.1 mg/L, lower than 3.4 ± 2.6 mg/L in the control group (*p* = 0.026). For peripheral blood inflammatory indicators, NLR in the treatment group was 2.4 ± 1.7, lower than 2.8 ± 1.6 in the control group, but the difference approached significance (*p* = 0.085); PLR (*p* = 0.139) and MLR (*p* = 0.157) showed no significant differences between groups (Fig. 1).

### Correlation Analysis

**Fig. 2.**
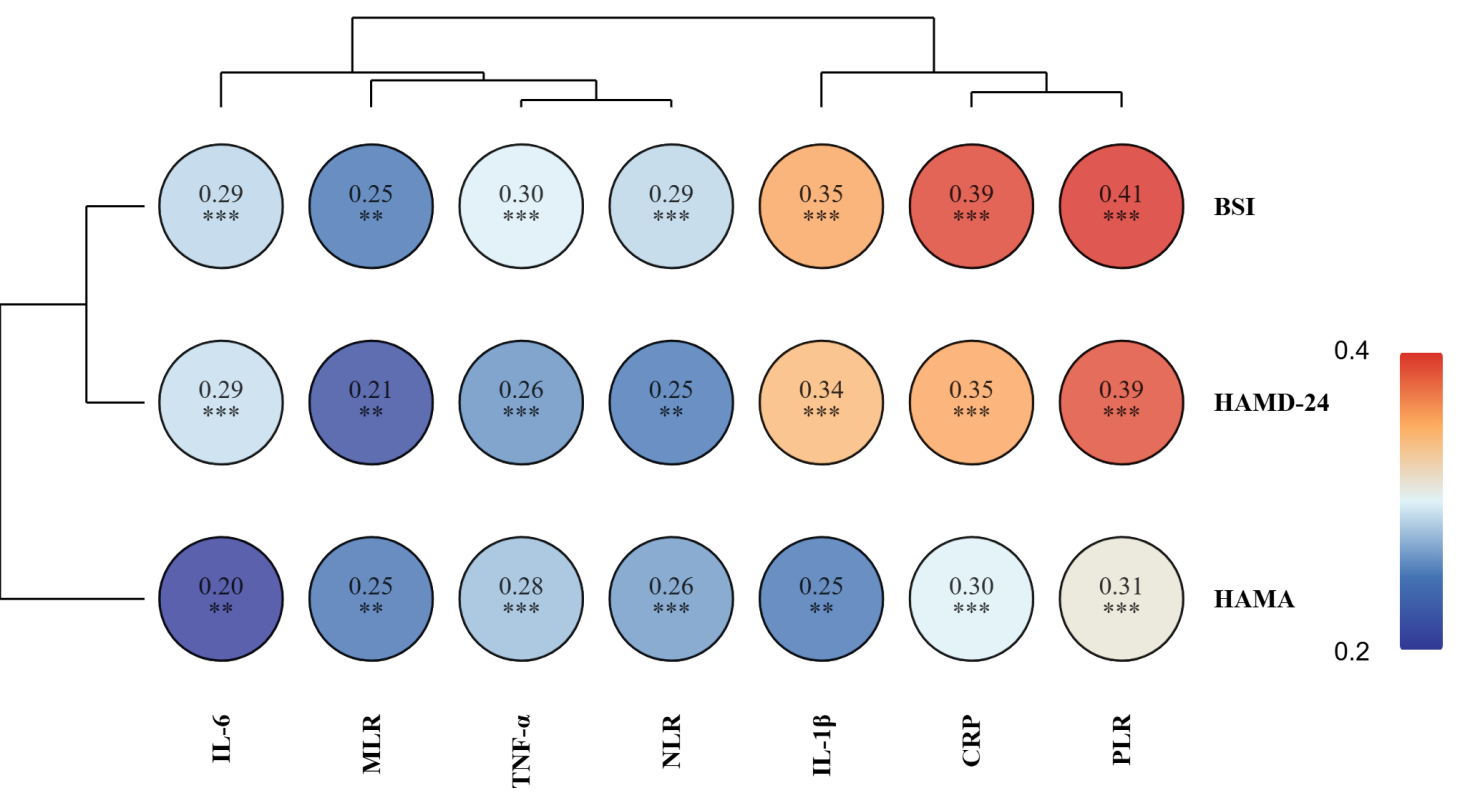
**Correlation heatmap between psychological scale scores and inflammatory markers after treatment**. HAMD-24, Hamilton Depression Rating Scale-24; HAMA, Hamilton Anxiety Rating Scale; BSI, Beck Scale for Sui detion-Chinesesion; IL-6, interleukin-6; IL-1β, interleukin-1β; TNF-α, tumor necrosis factor-α; CRP, C-reactive protein; PLR, platelet-to-lymphocyte ratio; NLR, neutrophil-to-lymphocyte ratio; MLR, monocyte-to-lymphocyte ratio. **p < 0.01; ***p < 0.001.

Spearman correlation analysis showed that BSI-CV (*r* = 0.29, 0.25, 0.30, 0.29, 0.35, 0.39, 0.41), HAMD-24 (*r* = 0.29, 0.21, 0.26, 0.25, 0.34, 0.34, 0.39) and HAMA (*r* = 0.20, 0.25, 0.28, 0.26, 0.25, 0.30, 0.31) were significantly positively correlated with all inflammatory factors (IL-6, IL-1β, TNF-α, CRP, PLR, NLR and MLR) after treatment. The BSI-CV showed the strongest correlations with inflammatory factors, followed by the HAMD-24. The HAMA showed relatively weak correlations (Fig. 2).

### Safety Evaluation

No significant difference in adverse reaction rate was found between the groups (*p* > 0.05). Main adverse reactions in the treatment group were mild gastrointestinal discomfort (12.5%), dizziness (7.5%) and insomnia (10.0%); in the control group, they were gastrointestinal discomfort (10.0%), dizziness (8.75%) and insomnia (12.5%). All adverse reactions were mild to moderate, with no serious adverse events.

## Discussion

This study systematically evaluated for the first time the efficacy of sertraline combined with intestinal microecological preparations in treating adolescent depression with suicidal ideation and its effects on inflammatory factors. The results indicate that combination therapy not only considerably improves clinical efficacy and ameliorates depression and anxiety symptoms but also effectively reduces serum inflammatory factor levels. This anti-inflammatory effect is closely related to clinical efficacy.

### Clinical Efficacy Advantages of Combination Therapy

The proportion of patients with severe depression in the combination therapy group was only 1.25%, which was far lower than 7.5% in the control group. This result suggests that combination therapy has clear advantages over monotherapy in alleviating depression severity. Notably, the present study found that the combined regimen considerably reduced the proportion of patients with moderate and severe depression (3.75% & 1.25% in the combination group vs. 13.75% & 7.5% in the monotherapy group). This shift in severity distribution may be mechanistically linked to the modulation of the gut–brain axis. The added probiotic may have enhanced intestinal barrier integrity, reduced systemic inflammatory tone and facilitated serotonergic and neurotrophic signalling, thereby not only improving overall response but also attenuating the neurobiological burden associated with severe depressive states. This finding aligns with the observed greater reductions in the levels of pro-inflammatory cytokines (IL-6, IL-1β and TNF-α) in the combination group, supporting the notion that gut-targeted adjunctive therapy can ameliorate key pathophysiological processes underlying depression severity.

Notably, this study particularly focused on suicidal ideation as an important clinical indicator. The BSI-CV scores were significantly positively correlated with the levels of various inflammatory factors (*r* = 0.25–0.41), and correlation coefficients were higher than those for the HAMD-24 and HAMA scores. These results suggest that inflammatory responses play an essential role in the neurobiological mechanisms of suicidal behaviour, consistent with the findings of Brundin *et al*. [23], who found significantly elevated inflammatory factor levels in the cerebrospinal fluid of people who attempted suicide and proposed the ‘inflammation-suicide’ hypothesis.

### Anti-inflammatory Mechanisms of Intestinal Microecological Modulation

This study observed significant decreases in the levels of multiple inflammatory factors in the combination therapy group, including IL-6, IL-1β, TNF-α and CRP. This systemic anti-inflammatory effect may be achieved through the following mechanisms:

First, probiotics, such as *B. subtilis* can restore gut microbiota balance, enhance intestinal barrier function and reduce translocation of bacterial endotoxins, including as lipopolysaccharides (LPSs), thereby lowering systemic inflammatory responses [24]. Patients with depression have increased intestinal permeability and elevated serum LPS levels, and LPSs are important triggers that activate peripheral and central inflammatory responses [25]. Second, probiotics can modulate the intestinal immune system by promoting regulatory T cell differentiation, increasing the production of anti-inflammatory cytokines, such as IL-10, while suppressing Th1/Th17 responses and reducing pro-inflammatory factor release [26]. The observed reduction in IL-1β is noteworthy, given its established role as a key product of NLRP3 inflammasome activation, which has been implicated in the pathogenesis of depression [27]. Third, the gut microbiota can communicate directly with the central nervous system through the vagus nerve, modulating HPA axis function and reducing stress responses and inflammation levels [28]. Animal experiments have confirmed that certain probiotic strains can reduce stress-induced cortisol elevation and pro-inflammatory factor expression [29].

### Study Limitations and Future Directions

A key limitation of this study is its retrospective cohort design. Treatment allocation was based on prescription records rather than randomisation, potentially introducing selection bias and limiting the strength of causal inference regarding the efficacy of the combination therapy. This study has the following additional limitations: (1) This has a retrospective design and thus lacks a placebo control group. It was not conducted in a double-blind manner, and thus measurement bias may have been introduced, particularly for subjective psychiatric scales, such as the HAMD-24. In addition, treatment allocation was not randomised, introducing potential for selection bias and confounding factors. (2) Relatively small sample size from a single centre, requiring caution in extrapolating results. (3) Observation period of only 12 weeks, requiring further study of long-term efficacy and relapse rates. (4) This study focused on evaluating the clinical efficacy and systemic inflammatory responses associated with the combined intervention. Although grounded in the gut-brain axis theory, no faecal microbiome analysis (e.g., 16S rRNA sequencing) was performed to directly confirm changes in microbial composition. Therefore, the observed reductions in inflammatory factors and improvements in symptoms may be interpreted as potentially related to intestinal microecological modulation, but specific mechanistic evidence regarding microbiota changes should be further validated in future studies. (5) Effects on cognitive function and quality of life were not assessed. (6) Furthermore, although sertraline dosing was flexible and followed the same titration protocol in both groups, the lack of dosestratified analysis limits the ability to fully separate the effects of the probiotic from those of dose variation. Future fixed-dose or dose-controlled designs are warranted to confirm the independent efficacy of probiotic adjunctive therapy.

Future research directions are as follows: (1) conducting prospective, multicentre, large-sample RCT studies to validate these findings; (2) extending follow-up time for the assessment of long-term efficacy and safety; (3) using metagenomic techniques in the analysis of changes in the gut microbiota and specific microbial mechanisms; (4) exploring difference in efficacy among different probiotic strains or combinations; (5) investigating biomarker-guided individualised treatment strategies.

## Conclusions

In this retrospective study, the use of sertraline combined with an intestinal microecological preparation was associated with significantly better outcomes compared to sertraline monotherapy in treating adolescent depression with suicidal ideation, effectively improving depression and anxiety symptoms while reducing serum inflammatory factor levels. Inflammatory factor levels are positively correlated with clinical symptom severity and showed potential use as exploratory biomarkers of treatment response. However, future studies are needed to establish validated cutoff values, sensitivity/specificity profiles and longitudinal predictive models before they can be considered reliable predictors in clinical practice. This combination therapy protocol has good safety profiles and provides new evidence-based medical evidence for comprehensive treatment of adolescent depression. In clinical practice, for adolescents with moderate depression and suicidal ideation, comprehensive treatment strategies combining antidepressants with intestinal microecological modulation may be considered a potential approach to improve efficacy and prognosis, pending further validation from larger prospective studies and individualised clinical assessment.

## Availability of Data and Materials

The datasets used and/or analysed during the current study were available from the corresponding author on reasonable request.
